# Diseases of the Eyes

**Published:** 1913-12

**Authors:** 


**Diseases of the Eyes.** By C. Devereux Marshall, F.R.C.S. Pp. xiii, 303. London: University of London Press. [1912]. 10s. 6d.

This is a text-book for students and practitioners who, although doing a certain amount of eye-work, cannot be considered experts.

It is a book full of knowledge, and also, which does not always follow, full of wisdom.

The subject is covered in a very comprehensive manner. It makes interesting reading, and unlike many text-books, it is not like a dictionary, so that it can be read with pleasure. The student is lucky who selects this book from which to learn his eye work.

There are articles written by Claud Worth, Bishop Harman and Harrison Butler on the various operations they have themselves devised. Probably this accounts for the fact that the picture of Worth's squint operation appears upside down as it did in his own book.

There is a very good chapter on colour vision, which is supervised by Edridge Green. It makes the subject clear and comprehensible to the ordinary medical reader.

We can confidently recommend the book.

